# γδ T Cells Are Reduced and Rendered Unresponsive by Hyperglycemia and Chronic TNFα in Mouse Models of Obesity and Metabolic Disease

**DOI:** 10.1371/journal.pone.0011422

**Published:** 2010-07-02

**Authors:** Kristen R. Taylor, Robyn E. Mills, Anne E. Costanzo, Julie M. Jameson

**Affiliations:** Department of Immunology and Microbial Science, The Scripps Research Institute, La Jolla, California, United States of America; University of Cambridge, United Kingdom

## Abstract

Epithelial cells provide an initial line of defense against damage and pathogens in barrier tissues such as the skin; however this balance is disrupted in obesity and metabolic disease. Skin γδ T cells recognize epithelial damage, and release cytokines and growth factors that facilitate wound repair. We report here that hyperglycemia results in impaired skin γδ T cell proliferation due to altered STAT5 signaling, ultimately resulting in half the number of γδ T cells populating the epidermis. Skin γδ T cells that overcome this hyperglycemic state are unresponsive to epithelial cell damage due to chronic inflammatory mediators, including TNFα. Cytokine and growth factor production at the site of tissue damage was partially restored by administering neutralizing TNFα antibodies *in vivo*. Thus, metabolic disease negatively impacts homeostasis and functionality of skin γδ T cells, rendering host defense mechanisms vulnerable to injury and infection.

## Introduction

Resident intraepithelial γδ T cells are responsible for maintaining epithelial integrity, regulating homeostasis and providing a first line of defense against pathogens and injury in mice and humans [1], [2], [3]. γδ T cells arise in the thymus during ontogeny and migrate, in waves, to epithelial tissues such as the skin, lung, intestine and reproductive tract where they populate these tissues for the life of the animal [4], [5]. In addition to their role in the innate immune response, γδ T cells regulate the subsequent recruitment of inflammatory cells to sites of injury and infection [6], [7], [8]. Murine skin resident T cells express a canonical Vγ3Vδ1 T cell receptor (TCR) and respond to a proposed, yet unknown, self antigen expressed by stressed or damaged keratinocytes [9], [10]. Skin γδ T cells display a dendritic morphology, retract their dendrites following activation and are critical for epidermal homeostasis and wound repair through their production of cytokines and regulation of inflammatory cells [1], [6], [11], [12], [13], [14], [15]. Mice deficient in γδ T cells exhibit disrupted skin homeostasis, impaired barrier function and delayed wound healing [1], [15], [16], [17]. In humans, the epidermis consists of a mixed resident αβ and γδ T cell population [18]. Similar to observations in mice, skin-resident Vδ1^+^ γδ T cells in humans produce cytokines and growth factors after activation and participate in wound repair [19].

In obesity and metabolic syndrome, the epidermal barrier is disrupted and skin complications can ultimately result in chronic and debilitating non-healing wounds and persistent infections [20]. Chronic wounds in obese and diabetic patients show diminished or altered levels of growth factors, impaired leukocyte infiltration and function and the absence of cell growth and migration over the wound [20]. Even with medical treatment, these chronic non-healing wounds may ultimately result in amputation of extremities [21]. Recent work has focused on the initiation of chronic inflammation in adipose tissue in obesity. An increase in effector CD8^+^ T cells and a decrease in CD4^+^ and T regulatory cells in adipose tissue have been shown to correlate with exacerbated adipocyte inflammation and metabolic disease progression [22], [23], [24]. However, the consequence of obesity and metabolic disease on the function of skin resident lymphocyte populations and how this contributes to skin complications associated with obesity and metabolic disease are unknown.

In this study we investigated how skin γδ T cell function becomes altered in obesity and metabolic disease. We show that the progression of metabolic disease impacts both the homeostasis and wound healing response of skin γδ T cells. Correlating with early hyperglycemia, the proliferation of skin γδ T cells is impaired, which ultimately results in a reduction in tissue-resident epidermal T cell numbers. The remaining skin γδ T cells overcome this hyperglycemic state, but exhibit altered metabolic and nutrient sensing pathways. The chronic inflammatory environment, specifically elevated TNFα, renders the remaining skin γδ T cells dysfunctional to tissue damage. In this inflammatory environment, skin γδ T cells are unresponsive to keratinocyte stimulation and unable to produce cytokines and epithelial regulating factors such as TGFβ1. We can improve skin γδ T cells function *in vivo* by blocking TNFα, providing evidence that chronic TNFα in metabolic syndrome contributes to skin γδ T cell dysfunction in wound healing.

## Results

### Skin γδ T cells are unable to maintain epidermal numbers in obesity

**Figure 1:**
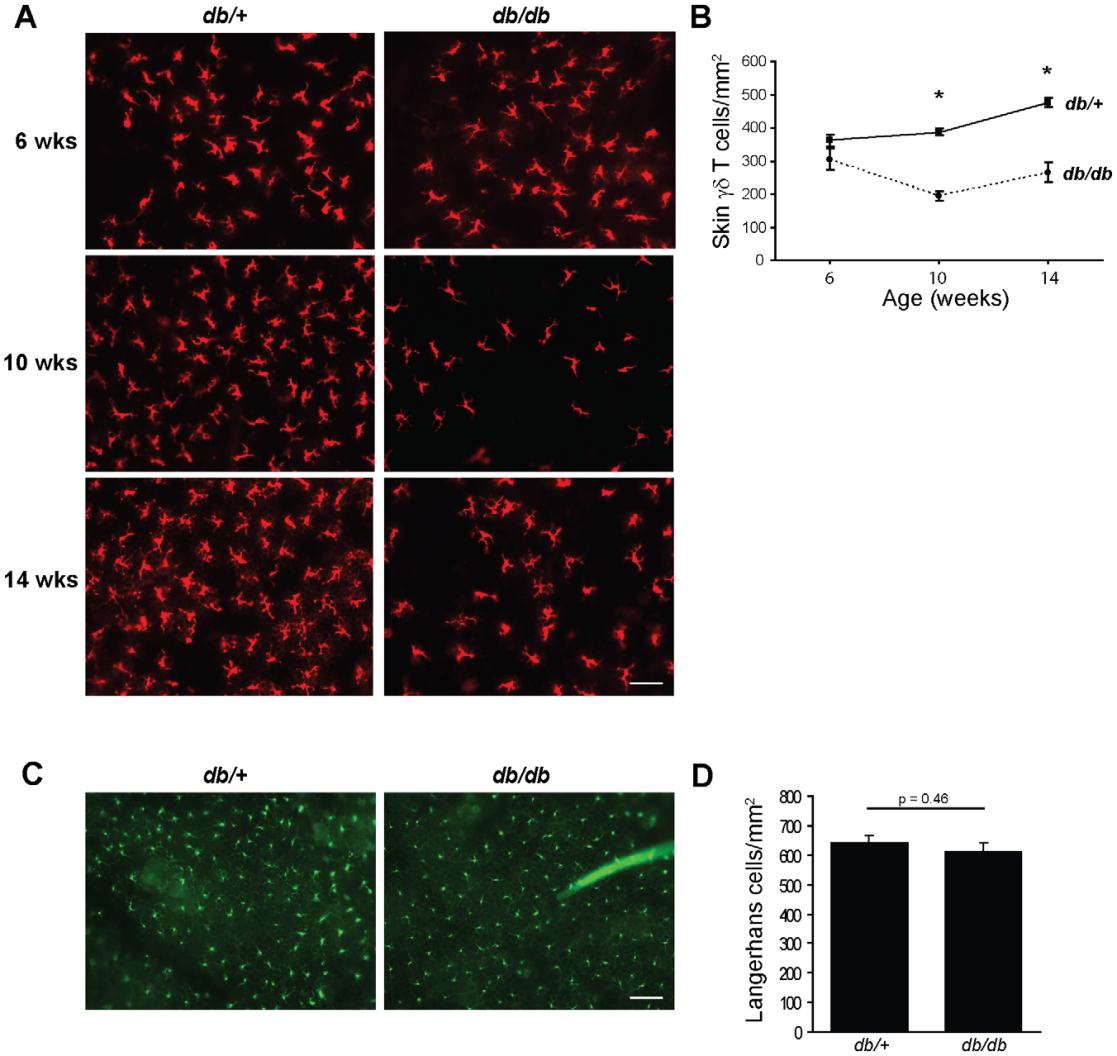
Reduced numbers of skin γδ T cells during obesity and metabolic disease is associated with hyperglycemia. (**A**) γδ TCR immunofluorescence staining of epidermal sheets from BKS *db/+* and *db/db* mice at 6-, 10- and 14-weeks of age. (**B**) Graphical representation of the number of epidermal γδ T cells in *db/+* (solid line) and *db/db* (dashed line) mice at each age. *p<0.005. (**C**) Epidermal sheets from 10-week old BKS *db/+* and *db/db* mice immunostained for the LC marker, langerin. (**D**) Graphical representation of the number of Langerhans cells at 10-weeks of age. All microscopy images were acquired at ×200. The bar represents 0.05 µm. Data (mean ± SEM) are representative of three independent experiments for each age group and a minimum of 15 fields per mouse.

Skin γδ T cells arise in the thymus during fetal development, migrate to the skin and actively expand to reach a maximum of ∼5% of the total cells in the epidermis. After this early migration, the epidermal skin γδ T cell compartment is maintained through self-renewal. To determine the impact of obesity and metabolic disease on skin γδ T cell survival and maintenance, we quantified γδ T cell numbers in epidermal sheets and analyzed their morphology starting at 6-weeks of age and continuing out to 14-weeks of age. Epidermal sheets from 6-week old *db/+* (lean control) and *db/db* mice demonstrated that skin γδ T cells seeded the epidermis, were present in expected numbers and exhibited their characteristic dendritic morphology (**Figure** **1A**). However, at this 6-week time point, a slight decrease in γδ T cell numbers was observed. By 8- and 10-weeks of age a pronounced decrease in skin γδ T cell numbers was apparent in obese *db/db* mice (**Figure** **1A and 1B**). Following this rapid decline, epidermal γδ T cells stabilized at 10-weeks of age and remained reduced out to 14-weeks of age (**Figure** **1A and 1B**).

In addition to the lymphocyte population, a resident dendritic cell population, the Langerhans cells (LC), also resides in the skin. To determine the impact of obesity and metabolic disease on another skin-resident immune population, we examined LC numbers using anti-langerin and anti-CD45.2 antibodies to stain epidermal sheets [25]. Obese *db/db* mice had similar numbers of LC in the epidermis as compared to lean *db/+* control mice at all ages tested (**Figure** **1C and 1D**). Our data suggest that the early progression of obesity and metabolic syndrome are marked by a selective inability of skin γδ T cells to maintain homeostatic numbers within the epidermis.

To address the possible contribution of leptin receptor deficiency on skin γδ T cells from *db/db* animals, we investigated the expression of leptin (Lep) and two leptin receptor isoforms (Lepr) in skin γδ T cells. No expression of either leptin or two leptin receptor isoforms, Ob-Ra and Ob-Rb, was detected in mRNA from skin γδ T cells isolated directly *ex vivo* or in the γδ 7–17 cell line *in vivo* (**Figure S1**).

### Hyperglycemia alters STAT5 signaling and impedes γδ T cell proliferation

**Figure 2:**
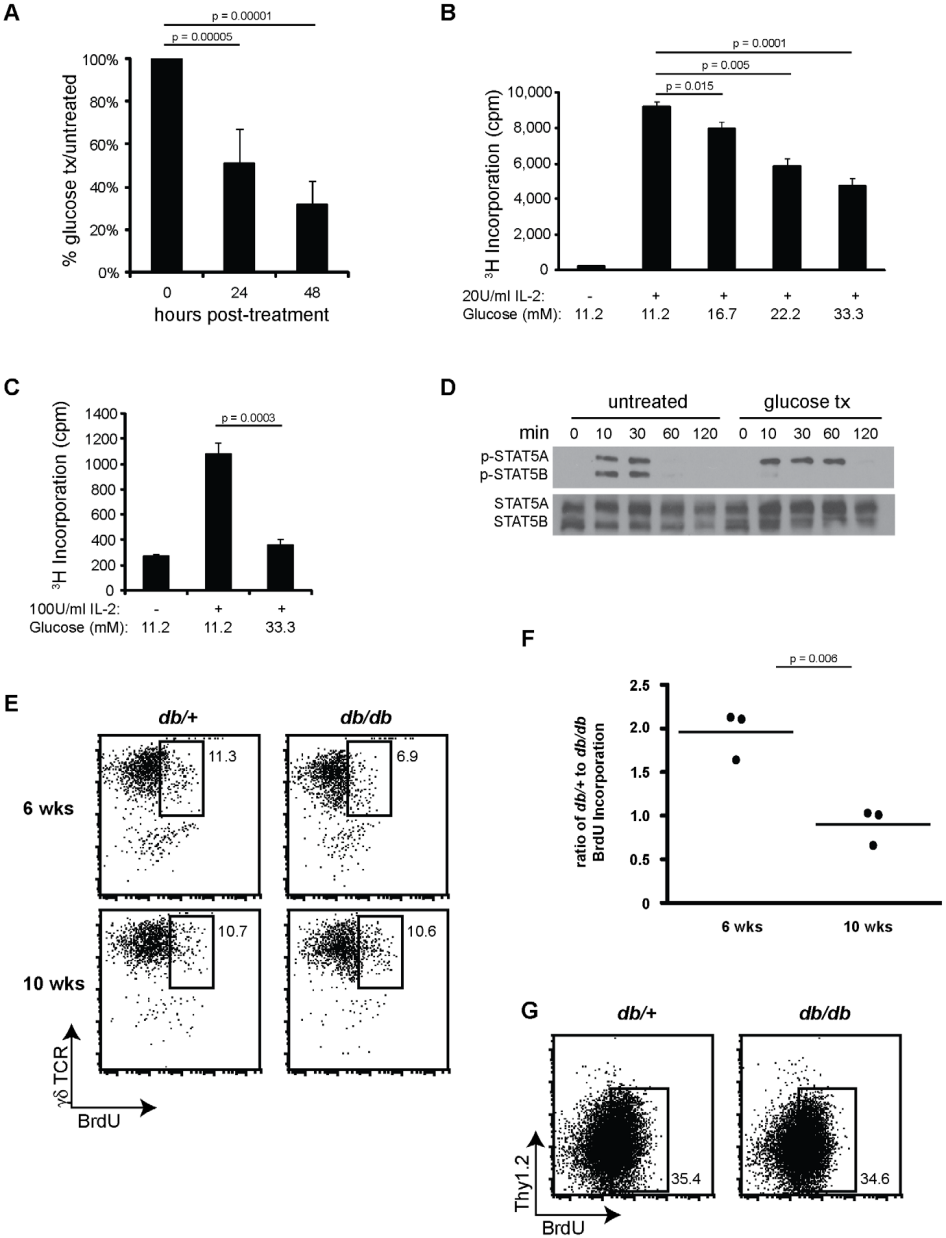
Regulation of skin γδ T cell proliferation by glucose is associated with decreased STAT5B phosphorylation. (**A**) *In vitro* growth of γδ 7–17 T cell line treated with 33.3 mM glucose at 0, 24 and 48 hours. Data (mean ± SD) presented as the % of glucose treated cells to untreated control cells. (**B, C**) Proliferation of (**B**) γδ 7–17 T cells and (**C**) freshly isolated γδ T cells sorted from wild-type B6 mice in IL-2 containing growth media supplemented with elevated glucose concentrations. Each experiment was performed in triplicate, data presented as mean ± SD. (**D**) The kinetics of expression of phosphorylated STAT5A and STAT5B following 40U/ml IL-2 stimulation in untreated and glucose treated γδ 7–17 T cells. Total STAT5 expression demonstrates even loading and expression. (**E**) Multiparameter flow cytometry of BrdU incorporation by skin γδ T cells isolated from 6- and 10-week old BKS *db/+* and *db/db* mice treated with BrdU for 7 days. The same number of events is presented for each dot plot, numbers indicate the percent of γδ T cells that have incorporated BrdU. Epidermal cells were gated on live Thy1.2^+^ events for γδ T cells. (**F**) Graphical representation of the ratio of BrdU incorporation by γδ T cells in BKS *db/+* to *db/db* mice at 6-weeks and 10-weeks of age, *n = 3* per strain and age. Shown are black dots to represent the ratio of each experiment, the black line represents the average of three experiments. (**G**) Multiparameter flow cytometry of BrdU incorporation by keratinocytes isolated from 6-week old BKS *db/+* and *db/db* mice treated with BrdU for 7 days. The same number of events is presented for each dot plot, numbers on the right indicate the percent of keratinocytes that have incorporated BrdU. Epidermal cells were gated on live γδ TCR^−^ events for keratinocytes. Data are representative of five (**A, B**) or three (**C**–**G**) separate experiments.

Between 6- and 10-weeks of age, BKS *db/db* mice are hyperglycemic and exhibit greater weight gain than their *db/+* control littermates (**Table S1**). To determine the impact of environmental factors that are present during this phase of disease, such as glucose and fatty acids, we tested whether the 7–17 skin γδ T cell line can maintain itself and survive when these factors are present and elevated. We found that 7–17 γδ T cells treated with 33.3 mM glucose resulted in a rapid decline of T cells within 24 to 48 hours of treatment (**Figure** **2A**). However, treatment of 7–17 γδ T cells with fatty acids did not inhibit γδ T cell growth (**Figure S2**).

To investigate the impact of glucose on skin γδ T cell proliferation, 7–17 cells were maintained in IL-2, treated with elevated glucose and proliferation determined. As shown in **Figure 2B**, there was a dose dependent inhibition of γδ T cell proliferation 36 hours post-glucose treatment. In addition to the 7–17 γδ T cell line, freshly isolated skin γδ T cells were sorted from epidermal cell preparations from wild-type mice, placed into IL-2 containing media in the presence of baseline (11.2 mM) or elevated (33.3 mM) glucose. Similar to observations with the 7–17 γδ T cell line, freshly isolated skin γδ T cells also displayed reduced proliferation in the presence of elevated glucose (**Figure** **2C**). This data suggests that skin γδ T cells are highly sensitive to elevations in glucose, affecting their ability to proliferate and maintain homeostatic numbers.

Since γδ T cells proliferate after stimulation with IL-2 in a glucose-sensitive manner, we next asked whether glucose treatment alters downstream IL-2 signaling. IL-2 receptor binding results in Jak1 and Jak3 activation, phosphorylation of STAT5 and translocation of the STAT5 complex to the nucleus where it regulates gene transcription [26]. Following stimulation of untreated skin γδ T cells with IL-2, phosphorylation of STAT5A and STAT5B peaked at 30 minutes, followed by a rapid decrease in phosphorylation (**Figure** **2D**). However, in glucose-treated γδ T cells, STAT5A was rapidly phosphorylated to peak levels within 10 to 30 minutes after IL-2 stimulation but displayed altered kinetics and prolonged phosphorylation compared to untreated cells. In addition, glucose-treated γδ T cells had negligible phosphorylation of STAT5B after IL-2 stimulation (**Figure** **2D**). This data suggests that diminished proliferation of skin γδ T cells may be due to altered IL-2 and STAT5 signaling in response to hyperglycemic conditions. Moreover, STAT5A/B signaling is critical to γδ T cell function as γδ T cells are absent in mice deficient in STAT5A/B [27].

To determine if diminished skin γδ T cell proliferation in BKS *db/db* mice accounts for the reduction in epidermal T cell numbers, we first had to investigate the rate of γδ T cell proliferation *in vivo*. Although long-lived, memory-like Vγ2^+^ T cells in the periphery have been shown to have very slow turnover [28], the rate of Vγ3^+^ T cell proliferation and homeostatic maintenance in the epidermis has yet to be defined. Unlike the rapid turnover of epithelial keratinocytes [29], [30], [31], LC turnover is much slower, between 5 and 10% of cells proliferating per week [32], [33]. To determine the rate of γδ T cell proliferation in the epidermis, control BKS *db/+* mice were treated for one week with BrdU in the drinking water and skin γδ T cells were analyzed for BrdU incorporation at 6- and 10-weeks of age. Skin γδ T cell proliferation in 6- and 10-week old lean *db/+* mice averaged approximately 11% of the total cells proliferating per week (**Figure** **2E, left panels**).

BrdU incorporation was then quantified in 6- and 10-week old BKS *db/db* mice to ascertain whether decreased proliferation accounts for diminished skin γδ T cell numbers in the *db/db* mouse. In contrast to the 10–12% BrdU incorporation of skin γδ T cells in 6-week old lean *db/+* mice, only half as many γδ T cells incorporated BrdU in *db/db* mice (**Figure** **2E, right panel, and 2F**). This reduced percentage of γδ T cells isolated from 6-week old *db/db* mice indicates decreased skin γδ T cell turnover in the BKS *db/db* mouse. Turnover of epithelial keratinocytes confirmed that BrdU was reaching the skin and being incorporated at a similar rate in 6-week old BKS *db/+* and *db/db* (**Figure** **2G**). In contrast to 6-week old mice, skin γδ T cells from obese 10-week old *db/db* mice had a similar percentage of skin γδ T cells incorporating BrdU as compared to control *db/+* mice (**Figure** **2E, right panel, and 2F**). This correlates with the data presented in **Figure 1B**, which shows that γδ T cell numbers stabilize in 10-week old *db/db* mice.

To confirm that skin γδ T cells were not undergoing increased apoptosis in BKS *db/db* mice, freshly isolated γδ T cells from the epidermis were stained with annexin-V and subject to propidium iodide incorporation (PI). No significant changes in skin γδ T cell annexin^+^PI^+^ populations were detected between lean *db/+* and obese *db/db* animals at multiple ages (**Figure S3A**). Furthermore, to verify that skin γδ T cells in the obese environment were not migrating out of the epidermis, whole skin cross-sections were stained with γδ TCR-specific antibodies and analyzed by immunofluorescent microscopy. We established that γδ T cells in the BKS *db/db* mouse remained localized to the epidermis and hair follicles (**Figure** **S3B**) and were not found migrating into the dermis. Additionally, skin-specific Vγ3^+^ T cells were not detected in lymph nodes providing further evidence that they have not migrated out of the epidermis (**Figure** **S3C**).

Taken together, this data demonstrates that hyperglycemia impacts skin γδ T cell proliferation, specifically at 6-weeks of age, ultimately reducing the population of skin γδ T cells in the epidermis by half. However, by 10-weeks of age, the remaining skin γδ T cells in the *db/db* animals have overcome the impaired proliferation induced by hyperglycemia.

### Skin γδ T cells are unresponsive to tissue damage in obesity

**Figure 3:**
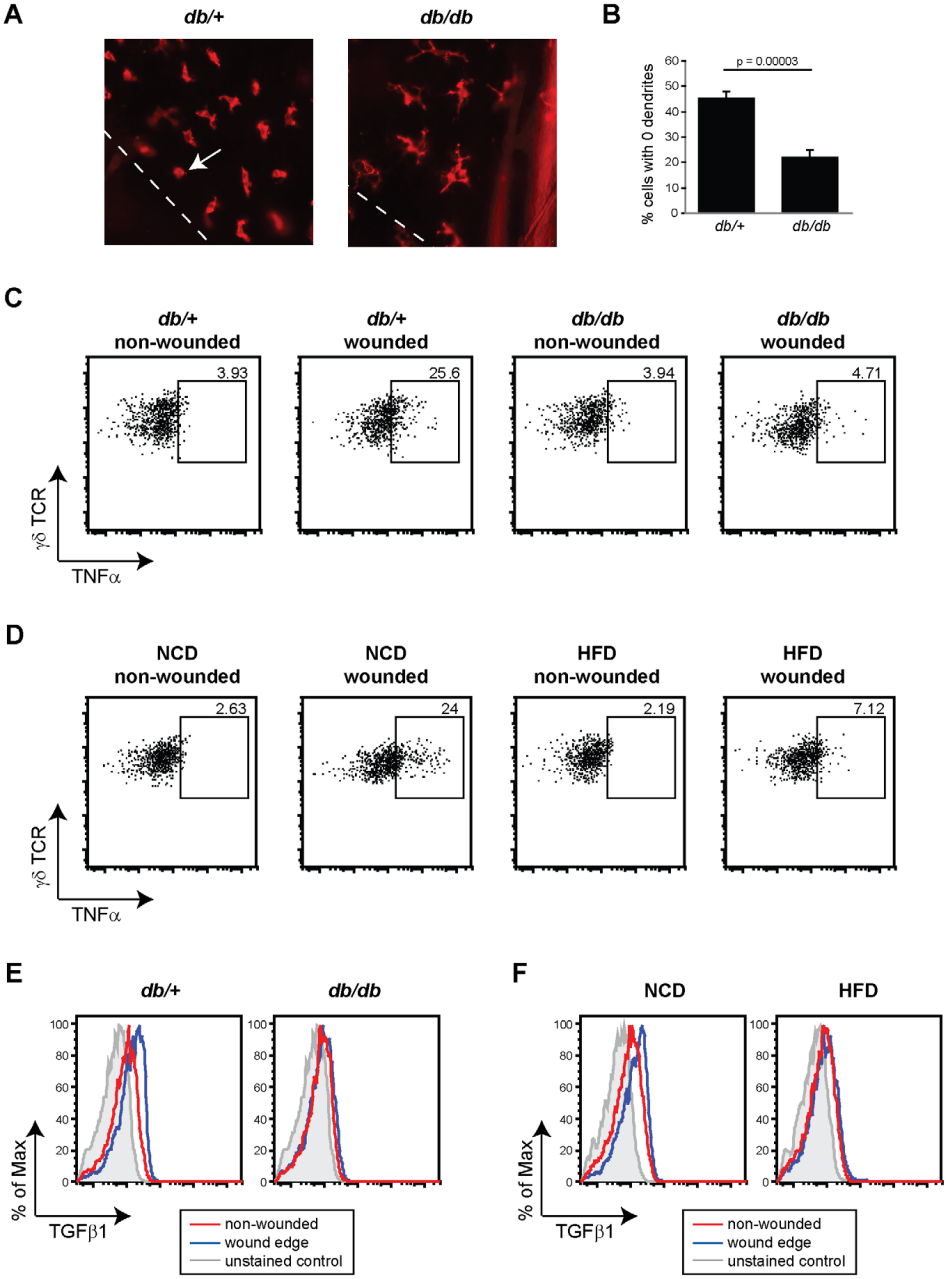
Obese mice display impaired skin γδ T cell wound healing functions after injury. (**A**) Immunofluorescent staining for γδ TCR in 12-week old lean BKS *db/+* and obese *db/db* mice four hours post-wounding. The white dashed line represents the wound edge and arrowheads depict γδ T cells that have rounded near the wound edge. A minimum of 10 images was acquired at the wound edge for each experiment and the number of dendrites was determined per cell, a minimum of 300 total cells were counted. (**B**) Shown is the percentage of γδ T cells with 0 dendrites per cell (mean ± SEM). All images were acquired at ×200 magnification. (**C, D**) Skin γδ T cell production of TNFα in non-wounded and wounded skin tissue of (**C**) 12-week old BKS *db/+* and *db/db* mice and (**D**) 27-week old B6 NCD and HFD mice. The numbers represent the percentage of cells expressing TNFα. Skin γδ T cell production of TGFβ1 in non-wounded and wounded skin tissue of (**E**) 12-week old BKS *db/+* and *db/db* mice and (**F**) 27-week old B6 NCD and HFD mice. The numbers represent the percentage of cells expressing TGFβ1. Epidermal cells were gated on live Thy1.2^+^ and γδ TCR^+^ to distinguish γδ T cells. Shown is one representative experiment, a minimum of three experiments were performed with similar results (**A**–**F**).

Since a population of skin γδ T cells survived the hyperglycemic environment, we next asked whether the remaining skin γδ T cells in the 10-week old *db/db* mice were able to respond to epithelial damage *in vivo*. One major function of skin γδ T cells is to recognize epithelial tissue damage and release cytokines and growth factors that facilitate wound repair. To investigate whether the remaining skin γδ T cells in the obese mouse are able to rapidly respond following injury, we monitored the ability of skin γδ T cells to retract their dendrites at the wound edge. Following injury and activation through their TCR, γδ T cells round-up at the wound edge and lose their dendritic morphology [1]. Cells distal to the wound site remain dendritic [1], confirming that this is a localized response to tissue damage. Full-thickness punch biopsy wounds were performed on obese 10- to 14-week old BKS *db/db* mice and skin γδ T cell morphology was examined at various time points by immunofluorescent microscopy. Our data indicates that skin γδ T cells in the obese *db/db* mice were delayed in their ability to round following wounding as compared to lean *db/+* control mice (**Figure** **3A**). These results were confirmed by quantifying the number of γδ T cells having retracted all their dendrites (**Figure** **3B**).

Another characteristic feature of skin γδ T cell activation is the upregulation of several Th1-type proinflammatory cytokines, including TNFα [34]. To determine if skin γδ T cells in obese mice have lost their ability to produce cytokines, we examined TNFα production by γδ T cells located along the wound edge. Full-thickness punch biopsy wounds were performed, γδ T cells were isolated from the wound edge, treated with brefeldin A and immediately stained using intracellular cytokine staining. This technique allows for the examination of skin γδ T cell function immediately *ex vivo* without any additional stimulus beyond the wound. In wild-type mice, cytokine production (using TNFα as a readout) was upregulated in skin γδ T cells directly adjacent to the wound site in control BKS *db/+* mice (**Figure** **3C**). However, γδ T cells isolated from the wounds of obese 10- to 14-week old BKS *db/db* mice did not produce TNFα (**Figure** **3C**).

We confirmed our *in vivo* wound healing results in another mouse model of obesity, the diet-induced obesity (DIO) model. C57BL/6J mice were started on a 60% kcal fat diet (B6 HFD) at 6 weeks of age compared to the normal chow diet (B6 NCD) (**Table S1**). As shown in **Figure 3D**, γδ T cells isolated from B6 NCD mice upregulated TNFα production at the wound edge compared to non-wounded controls. However, similar to BKS *db/db* animals, skin γδ T cells isolated from 26- to 32-week old B6 HFD mice had little upregulation of TNFα at the wound edge (**Figure** **3D**).

In addition to early release of proinflammatory molecules, growth factor production is another key function of skin γδ T cells in response to epithelial damage. We therefore reasoned that this functional response of skin γδ T cells was likely to be disrupted in skin γδ T cells in the obese environment. To address this directly, we investigated intracellular TGFβ1 production in skin γδ T cells isolated from wounded lean control and obese animals. Skin γδ T cells from control 10- to 14-week old BKS *db/+* animals increased TGFβ1 production at the wound edge 24 hours post-wounding (**Figure** **3E**). However, skin γδ T cells from obese 10- to 14-week old *db/db* mice had little to no upregulation in TGFβ1 expression (**Figure** **3E**). This defective TGFβ1 production was confirmed in our second model of obesity, the DIO model. Skin γδ T cells isolated from the wound edge of B6 NCD mice upregulated TGFβ1 production, however, skin γδ T cells isolated from the wound edge of 26- to 32-week old B6 HFD mice had impaired TGFβ1 upregulation (**Figure** **3F**). Therefore, in addition to defective cytokine production, skin γδ T cells in obesity and metabolic disease were unable to upregulate TGFβ1 production at the wound edge, an important growth factor in several aspects of wound repair.

Delayed rounding and the inability of skin γδ T cells to produce cytokines at the wound edge only occurred in obese 10- to 14-week old *db/db* animals. Skin γδ T cells in 6-week old *db/+* and *db/db* mice retracted their dendrites similarly within 4 hours post wounding and were able to upregulate TNFα adjacent to the wound edge (**data not shown**). Together, this data suggests two separate stages of disease: 1) an early defect in skin γδ T cell proliferation due to hyperglycemia that eventually results in half the number of skin γδ T cells residing in the epidermis and 2) a later defect characterized by the inability of skin γδ T cells to perform tissue repair functions *in vivo*.

### Impaired skin γδ T cell nutrient sensing and activation in obesity

The inability of skin γδ T cells to be activated and produce cytokines and growth factors following epithelial damage occurred only in 10- to 14-week old BKS *db/db* and not 6-week old mice. This unresponsive state was not caused by hyperglycemia and suggests that other environmental factors, such as chronic inflammatory factors, or cell-intrinsic factors may be responsible for the lack of tissue damage responses. To better understand the impact of metabolic disease on skin γδ T cells, we performed microarray analysis on skin γδ T cells sorted from total epidermal cell preparations from 10-week old BKS lean *db/+* and obese *db/db* mice.

**Figure 4:**
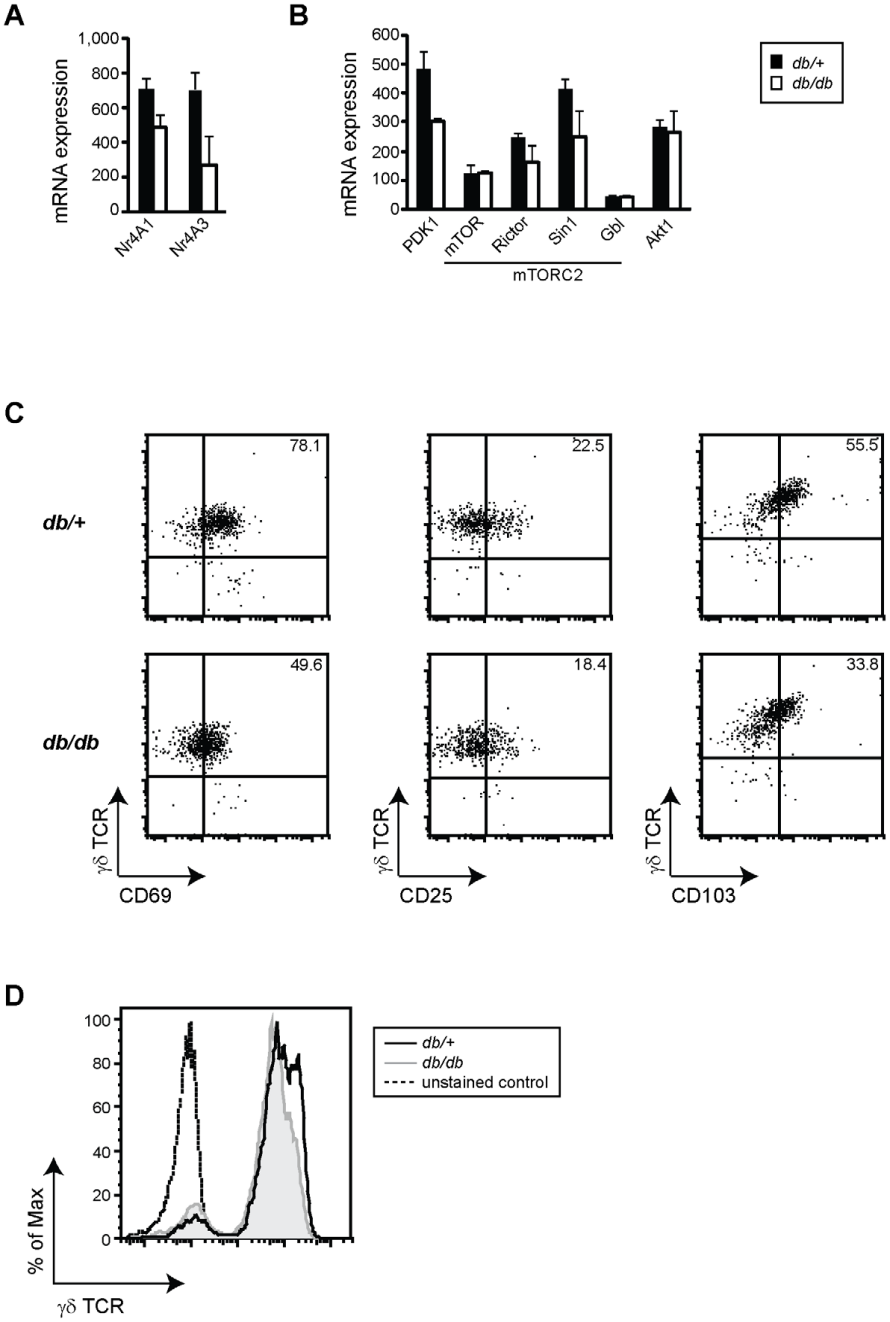
Impaired activation and nutrient sensing by skin γδ T cells in obese mice. (**A, B**) Microarray analysis of skin γδ T cells isolated from 10-week old BKS *db/+* and *db/db* mice. Shown is gene expression of molecules associated with (**A**) insulin sensitivity and (**B**) PI3K/Akt/mTOR signaling. Data is presented as the mean of two independent experiments ± SEM. **(C)** Multiparameter flow cytometry of CD69, CD25 and CD103 on the cell surface of γδ T cells isolated from BKS *db/+* and *db/db* in mice at 10-weeks of age. Numbers in the top right corners indicate percent of γδ T cells. (**D**) γδ TCR expression on γδ T cells isolated from BKS *db/+* (solid line) and *db/db* (shaded gray) at 10-weeks of age. Dotted lines represent unstained controls. Epidermal cells were gated on live Thy1.2^+^ to distinguish γδ T cells. A minimum of three experiments were performed per age, shown is one representative experiment for each, the same number of events is presented for each dot plot.

Based on the gene array, we found that skin γδ T cells differentially express NR4A1 and NR4A3, two orphan nuclear receptors which have been shown to sensitize muscle to insulin and have been reported to be underexpressed in obesity and type 2 diabetes [35]. We observed reduced expression of both NR4A1 and NR4A3 in γδ T cells isolated from obese *db/db* mice (**Figure** **4A**), suggesting that skin γδ T cells residing in *db/db* animals have decreased insulin sensitivity. Additionally, Pdk1, a central molecule that regulates Akt function, and two members of the mTORC2 complex, Rictor and Sin 1 (Mapkap1), all display decreased gene expression in skin γδ T cells isolated from obese *db/db* mice (**Figure** **4B**). Together these genes, which are necessary for the growth and function of γδ T cells [36], were altered in obese mice and reveal a breakdown in the normal signaling pathways required for skin γδ T cells homeostasis and function.

A breakdown in skin γδ T cell signaling pathways may result in changes to their characteristic innate T cell phenotype and function. Skin γδ T cells express a Vγ3Vδ1 TCR and constitutively elevated levels of the activation markers CD69 and CD25 (IL-2 receptor α), suggesting that they are primed to rapidly respond to TCR-mediated activation and growth factors, such as IL-2 [1], [36], [37]. No changes were observed in the expression of activation markers on skin γδ T cells isolated from 6-week old BKS *db/+* and *db/db* mice (**Figure S4A**). However, in obese *db/db* mice at 10-weeks of age, skin γδ T cells reproducibly displayed diminished levels of CD69, CD25 and CD103 (**Figure** **4C**). Furthermore, γδ TCR expression was reproducibly decreased in 10-week old obese *db/db* mice (**Figure** **4D**), but no decrease in γδ TCR expression was observed in 6-week old *db/db* mice (**Figure S4B**). Decreased expression of activation markers and γδ TCR may be due to overstimulation by stressed keratinocytes in obesity and metabolic disease.

### γδ TCR does not contribute to epidermal T cell dysfunction in obesity

**Figure 5:**
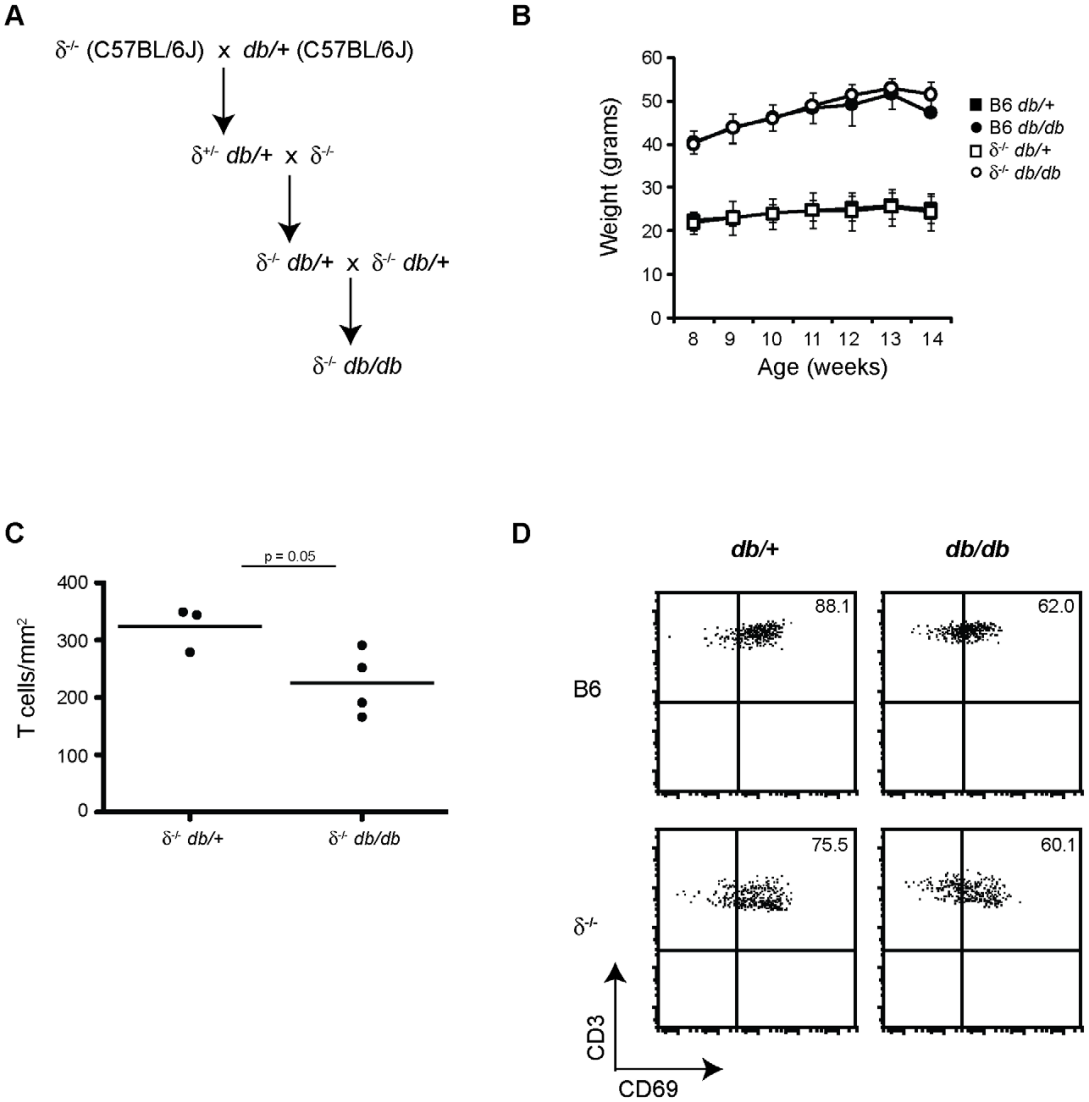
γδ TCR does not contribute to defective skin γδ T cells in obese mice. (**A**) δ^−/−^ *db/db* mice were generated by breeding C57BL/6J δ^−/−^ mice with C57BL/6J *db/+* mice. (**B**) Weight of δ^−/−^ *db/db* and B6 *db/db* obese mice compared to their δ^−/−^ *db/+* and B6 *db/+* lean littermates. Data is presented as the mean weight ± SD. Between two and eleven mice were weighed per age per strain. (**C**) Graphical representation of the number of epidermal T cells at 14-weeks of age. Skin γδ T cells were counted in epidermal ear sheets from three δ^−/−^ *db/+* mice and four δ^−/−^ *db/db* mice. The mean was determined for each experiment (black dots) and the black line represents the average of all the experiments. A minimum of 15 fields were counted for each mouse per experiment, with a minimum of 500 cells per experiment, a minimum of three independent experiments were performed. (**D**) Multiparameter flow cytometry of CD69 expression on the cell surface of γδ T cells isolated from B6 *db/+* and *db/db* and δ^−/−^ *db/+* and *db/db* mice at 14-weeks of age. Numbers on the top right corner indicate percent of αβ T cells. Epidermal cells were gated on live CD3^+^ and Thy1.2^+^ to distinguish epidermal T cells. A minimum of three experiments were performed, shown is one representative experiment, the same number of events is presented for each dot plot.

To investigate the contribution of the γδ TCR to the hyporesponsive state of skin γδ T cells in obesity, we crossed B6 δ^−/−^ and B6 *db/+* animals to generate mice lacking γδ TCR that develop obesity and metabolic disease (**Figure** **5A**). The epidermis of γδ T cell knockout mice (δ^−/−^) lacks Vγ3^+^ T cells but does have an αβ T cell population that takes up residence, however, these αβ T cells do not respond to keratinocyte damage [37]. No differences in breeding, litter size or growth of the animals were observed in the B6 δ^−/−^ *db/db* mice as compared to B6 *db/db* animals. Both male and female B6 δ^−/−^ *db/db* mice gained weight and became obese similar to B6 *db/db* mice (**Figure** **5B**).

To determine the impact of the γδ TCR on maintenance of homeostatic numbers of epidermal T cells in δ^−/−^ *db/db* animals, epidermal αβ T cells were visualized using immunofluorescent microscopy. In both δ^−/−^ *db/+* control and δ^−/−^ *db/db* mice, the only T cell population in the epidermis was CD3^+^ αβ T cells; no γδ T cells or other CD3^+^ populations were present, similar to the epidermal T cell makeup of B6 δ^−/−^ mice (**data not shown**). However, in 14-week old obese δ^−/−^ *db/db* mice there were ∼30% fewer epidermal αβ T cells compared with lean δ^−/−^ *db/+* control animals (**Figure** **5C**). This suggests that the keratinocyte antigen-specific γδ TCR is not necessary for the decline in epidermal T cell numbers observed in obesity.

Although the epidermal αβ T cells identified in B6 δ^−/−^ mice are not responsive to keratinocyte damage, they do express the activation markers CD69, CD25 and CD103 similar to γδ T cells in the skin [37]. Since expression of these molecules was diminished on γδ T cells in the obese environment, we determined whether activation markers on epidermal αβ T cells in the B6 δ^−/−^ *db/db* mouse were similarly affected. Decreased expression of both CD69 (**Figure** **5D**) and CD25 (**data not shown**) was observed on epidermal αβ T cells in 14-week old obese B6 δ^−/−^ *db/db* mouse similar to that observed on epidermal γδ T cells in obese B6 *db/db* mice. Together, this data suggests that the hyporesponsiveness observed in γδ T cells of obese mice is not TCR mediated or a direct consequence of overactivation by stressed keratinocytes. Therefore, dysfunction of skin γδ T cells in obesity and metabolic disease may be a direct consequence of the inflammatory milieu of the obese environment.

### Rescue of skin γδ T cell function ex vivo

**Figure 6:**
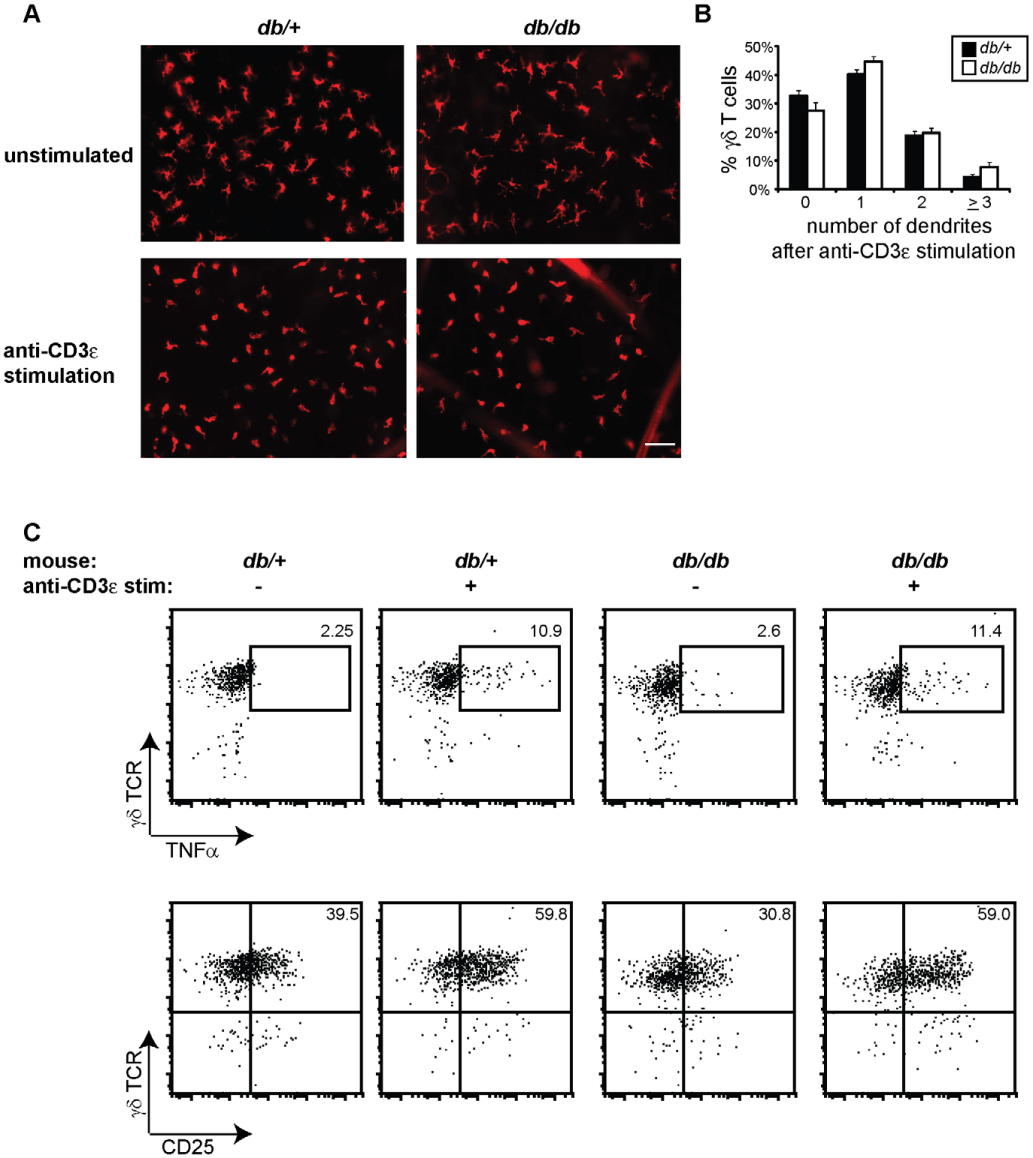
The obese environment inhibits skin γδ T cell function. (**A**) Skin γδ T cell morphology changes in epidermal sheets isolated from 10- to 14-week old BKS *db/+* and *db/db* following *in vitro* stimulation with 10 µg/ml anti-CD3ε antibody compared to unstimulated control. All microscopy images were acquired at ×200 and the bar represents 0.05 µm. (**B**) Shown is a graphical representation of the percentage of skin γδ T cells with 0, 1, 2 or ≥3 dendrites, which represent the degree of γδ T cell rounding (mean ± SEM), in epidermal ear sheets from 10- to 14-week old BKS *db/+* and obese *db/db* animals stimulated with 10 µg/ml anti-CD3ε antibody. Three independent experiments were performed, a minimum of 10 fields were counted for each, and this data represents the average of all 35 fields and approximately 1000 total cells. (**C**) Multiparameter flow cytometry of TNFα production and CD25 expression by γδ T cells isolated from 10- to 14-week old BKS *db/+* and *db/db* mice following overnight stimulation with 1 µg/ml anti-CD3ε. Numbers in the upper right corner indicate percent γδ T cells. Epidermal cells gated on live Thy1.2^+^ events. Data are representative of at least three independent experiments.

If the environment in obesity and metabolic disease contributes to skin γδ T cell dysfunction, we hypothesized that removal from this environment would improve skin γδ T cell function. To investigate whether the response of skin γδ T cells in obese mice can be restored by removal from their environment, we isolated epidermal sheets from 10- to 14-week old obese BKS *db/db* mice and lean *db/+* controls and stimulated the skin-resident T cells *in vitro* with anti-CD3ε antibody. After 6 hours in culture, we visualized epidermal sheets by immunofluorescent microscopy and quantified the number of dendrites per cell to determine cellular rounding after stimulation. The majority of γδ T cells in unstimulated epidermal sheets exhibited 3 or more dendrites per cell (**Figure** **6A**). However, after anti-CD3ε stimulation, γδ T cells began to round up similarly in epidermal sheets isolated from obese *db/db* and control *db/+* mice, as indicated by the reduced number of skin γδ T cells with 3 or more dendrites per cell (**Figure** **6A and 6B**). This indicates that removing epidermal cells from the obese *db/db* mouse, where they were unable to round upon wounding, restores the ability of γδ T cells to respond to stimulation.

Since stimulating epidermal sheets from obese *db/db* mice *ex vivo* restored the ability of skin γδ T cells to round, we next identified whether other γδ T cell functions could be rescued as well. To determine if cytokine production could be restored by removing γδ T cells from the obese environment, we isolated epidermal cells from 10- to 14-week old BKS lean *db/+* and obese *db/db* mice and cultured them in plates either coated with PBS (unstimulated) or anti-CD3ε antibody. Strikingly, upon removal from the obese environment, anti-CD3ε stimulated skin γδ T cells from obese *db/db* mice were able to produce cytokines, such as TNFα, and upregulate the activation marker CD25 to a similar degree as skin γδ T cells isolated from control *db/+* animals (**Figure** **6C**). Together, these data demonstrate that the dysfunction of skin γδ T cells in the obese *db/db* mouse is not permanent. It suggests that by removing extrinsic factors present in obesity and metabolic disease through the isolation of these cells from the epidermis, the hyporesponsive state of skin γδ T cells can be reversed.

### Blocking TNFα in obese mice restores skin γδ T cell function in epithelial repair

**Figure 7:**
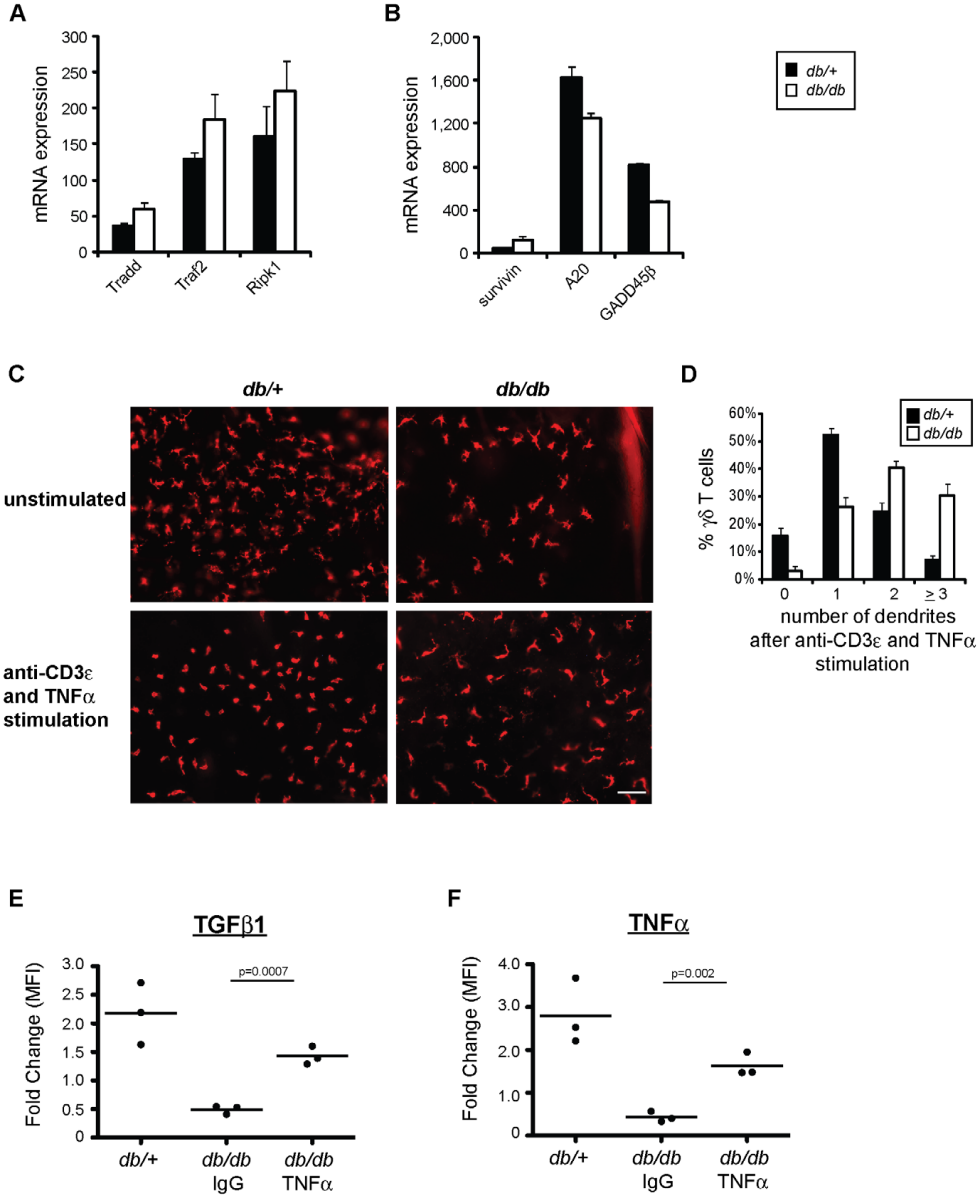
Neutralization of TNFα rescues skin γδ T cell function at the wound site. (**A, B**) Microarray analysis of skin γδ T cells isolated from 10-week old BKS *db/+* and obese *db/db* mice. Shown is gene expression of molecules associated with TNFα signaling. Data is presented as the mean of two independent experiments ± SEM. (**C**) Epidermal sheets isolated from 10- to 14-week old BKS *db/+* and obese *db/db* animals either unstimulated or stimulated with 10 µg/ml anti-CD3ε antibody and 100 ng/ml TNFα. All microscopy images were acquired at ×200 and the bar represents 0.05 µm. (**D**) Quantification of the percentage of skin γδ T cells with 0, 1, 2 or ≥3 dendrites, which represent the degree of γδ T cell rounding (mean ± SEM), in epidermal ear sheets from 10- to 14-week old BKS *db/+* and obese *db/db* animals stimulated with 10 µg/ml anti-CD3ε antibody and 100 ng/ml TNFα. Three independent experiments were performed, a minimum of 10 fields were counted for each, and this data represents the average of all 35 fields and approximately 1000 total cells. (**E, F**) Fold change in MFI of (**E**) TGFβ1 and (**F**) TNFα expression in skin γδ T cells isolated from the wound edge compared to non-wound edge cells. Skin γδ T cells from 10- to 14-week old BKS *db/+* mice were used as a positive control, 10- to 14-week old *db/db* mice were either treated with 1 mg/kg IgG control antibody or anti-TNFα antibody for a minimum of four days. Shown in fold change in MFI for 3 separate experiments, significance was determined by *t-test*.

Increased plasma TNFα levels correlate with obesity and insulin resistance in both humans and animals [38]. Our microarray data revealed that several members of the TNFα signaling pathway were increased in skin γδ T cells isolated from obese *db/db* mice, including Traf2, Tradd and Ripk1 (**Figure** **7A**), which lead to activation of NF-κB and Jun N-terminal kinase (JNK) [39]. As shown in **Figure 7B**, downstream molecules contributing to survival, such as Birc5 (survivin), are increased in skin γδ T cells isolated from obese *db/db* animals. However, molecules that negatively regulate Ripk1 and Jnk signaling, such as Tnfaip3 (A20) and GADD45β respectively [40], are decreased in γδ T cells isolated from obese *db/db* mice.

Since elevated gene expression of TNFα signaling molecules was observed in skin γδ T cells isolated from obese *db/db* mice, we set out to determine the consequence of elevated and chronic TNFα levels on skin γδ T cell function. Exogenous TNFα was added to cultured epidermal sheets isolated from control and obese animals. If TNFα alone contributes to the suppressive inflammatory milieu, skin γδ T cells would remain impaired upon *ex vivo* stimulation in the presence of this cytokine. Epidermal sheets from 10- to 14-week old lean *db/+* mice, which have not been exposed to chronic TNFα in their environment, rounded when stimulated with anti-CD3ε antibody in the presence of acute TNFα (**Figure** **7C and 7D**). However, γδ T cells in epidermal sheets isolated from obese *db/db* animals, which have been exposed to elevated and chronic TNFα in their environment, displayed delayed rounding when stimulated with anti-CD3ε antibody only if TNFα was present (**Figure** **7C and 7D**). As shown in **Figure 6A**, epidermal sheets from obese *db/db* mice were able to round following stimulation with anti-CD3ε antibody alone. This suggests that TNFα alone alters the ability of skin γδ T cells to round following stimulation, providing a mechanism for skin γδ T cell dysfunction.

Due to the contribution of chronic TNFα to γδ T cell dysfunction, we investigated whether skin γδ T cell responses to tissue damage could be restored *in vivo* by treatment with neutralizing anti-TNFα antibody. 10- to 14-week old obese *db/db* animals were treated daily for a minimum of four days with 1 mg/kg anti-TNFα or IgG control antibody. On day 4, full-thickness punch biopsy wounds were performed on each animal and epidermal cells were isolated around the wound edge 24 hours post-wounding. Skin γδ T cells isolated from obese *db/db* animals treated with anti-TNFα antibody showed improved TGFβ1 production as compared to *db/db* animals treated with IgG control antibody (**Figure** **7E**). Similar rescue of TNFα production was observed in skin γδ T cells isolated from *db/db* animals treated with anti-TNFα (**Figure 7F**). A significant improvement in skin γδ T cell function at the wound site suggests that chronic inflammatory conditions, specifically in the form of TNFα, contributes to skin γδ T cell hyporesponsiveness to *in vivo* wounding in obesity and metabolic disease.

## Discussion

Skin γδ T cells contribute to homeostatic maintenance of the epidermis and respond early to epithelial damage. Skin complications associated with obesity, metabolic disease and type 2 diabetes include barrier dysfunction, chronic non-healing wounds and increased infection. Due to their role in epidermal homeostasis and early response to keratinocyte damage, we investigated whether skin γδ T cell are functional in mouse models of obesity and metabolic disease. Strikingly, we observed a biphasic progression of epidermal T cell dysfunction and the parameters responsible for each phase of T cell dysfunction were distinct. Hyperglycemia impacted early skin γδ T cell proliferation and homeostasis, ultimately resulting in reduced epidermal T cell numbers. Chronic inflammation, occurring later in metabolic disease, rendered skin γδ T cells hyporesponsive to *in vivo* stimulation. In spite of this, skin γδ T cell dysfunction was reversible as improved cytokine production to *in vivo* stimulation was restored by systemic anti-TNFα antibody treatment. To our knowledge, this is the first description correlating different stages of lymphocyte dysfunction to disease progression in obesity.

Nutrients, such as glucose, are critical for lymphocyte survival, proliferation, differentiation and function [41], [42]. Many growth factors, such as insulin, IGF-1 and members of the common γ_c_ cytokine family (IL-2, IL-4, IL-7, IL-15) increase glucose uptake and metabolism via signaling through the PI3K/Akt pathway [42]. For example, IL-7 signaling in lymphocytes results in STAT5 and PI3K/Akt activation-induced glucose uptake [43]. However, we report here that during the first phase of dysfunction, skin γδ T cells are highly susceptible to alterations in glucose concentrations. Similarly, both αβ T cells and B cells have been shown to exhibit reduced proliferation when exposed to elevated glucose concentrations *in vitro* [43]. This suggests that although glucose and other nutrients may be critical for lymphocyte homeostasis and function, a chronic overabundance of nutrients is detrimental to the maintenance of γδ T cells in the epidermis.

Elevated glucose resulted in altered STAT5 phosphorylation after IL-2 stimulation *in vitro* and ultimately impaired γδ T cell proliferation. STAT5A/B signaling is critical to γδ T cells as mice deficient in STAT5A/B lack γδ T cells [27]. The inability of glucose-treated γδ T cells to phosphorylate STAT5B in response to IL-2 points directly to an effect on proliferation as mice expressing a constitutively active STAT5B have an expanded γδ T cell population [44]. Additionally, the severity of loss of skin γδ T cells in BKS *db/db* mice correlates with a period of rapid expansion of γδ T cells in the epidermis at 6-weeks of age. The hyperglycemic conditions during this seeding are severe in BKS *db/db* mice which may explain the sharp decrease in γδ T cells. Overall, these data demonstrate that skin γδ T cells are highly sensitive to metabolic changes, such as hyperglycemia, in the cellular environment and respond to this stress by shutting down nutrient sensing pathways, such as cytokine and growth factor signal reception, resulting in decreased homeostatic proliferation and a reduced epidermal T cell compartment.

In the next phase of metabolic disease, skin γδ T cells become unresponsive to tissue damage, resulting in reduced production of skin γδ T cell cytokines and growth factors. Skin γδ T cells are important mediators of inflammation and tissue repair as mice deficient in γδ T cells (δ^−/−^ mice) exhibit delayed wound healing [1]. Additionally, skin-resident T cells in chronic wounds isolated from human patients do not upregulate growth factor production, which may contribute to the inability of chronic non-healing wounds to resolve [19]. In addition to the production of cytokines by skin γδ T cells early in tissue damage, skin γδ T cells also produce growth factors which are critical to skin homeostasis [16]. We observed a decrease in homeostatic TGFβ1 production by skin γδ T cells and an inability to upregulate TGFβ1 following injury in obesity and metabolic disease. In the skin, the effects of TGFβ1 are broad and contribute to various aspects of wound healing including inflammation, angiogenesis, tissue remodeling and reepithelialization [45]. Altered TGFβ1 production by skin γδ T cells in obesity and metabolic disease may impact multiple phases of epidermal homeostasis and early and late stages of tissue repair.

To understand how the environment impacts the ability of skin γδ T cells to respond to *in vivo* damage, we performed microarray analysis to investigate alterations in gene expression and found an increase in expression of molecules involved in TNFα signaling. In primary cells, TNFα induces NF-κB but not cell death pathways, and chronic TNFα would predictably result in chronic NF-κB activation, gene expression and survival [39]. This persistent activation leads to the induction of reactive oxygen species [39], which can attenuate T cell responses [46], [47], and uncouple TCR signal transduction, resulting in lower cell surface expression of the TCR/CD3 complex [48]. This supports our observation that cell surface γδ TCR expression is decreased and downstream molecules regulating survival and negative feedback of NF-κB signaling were altered in skin γδ T cells isolated from obese mice.

In addition, chronic TNFα and persistent NF-κB activation negatively impact other cell signaling pathways, including PI3K/Akt/mTOR signaling [36], [49], [50]. TNFα and NF-κB suppress TSC1 inhibition of mTORC1, resulting in hyperactive mTORC1 activity, which contributes to insulin resistance [50]. Recently, mTORC1 has been shown to negatively inhibit mTORC2 signaling, a necessary complex for Akt activation, and may negatively inhibit growth factor signaling in pathways that don't require IRS-1 [51]. Furthermore, knockdown or deletion of mTORC2 complex molecules, including Rictor, Sin1 and Gbl, result in defective mTORC2 complex assembly and Akt activation [52], [53], [54]. Both mTORC1 and mTORC2 have been shown to be critical for skin γδ T cell homeostasis and *in vivo* wound healing response [36]. Chronic TNFα stimulation of skin γδ T cells results in direct effects, including alterations in TCR expression, and effects on other signaling pathways, including mTOR and Akt. These alterations in signaling ultimately render epidermal T cells hyporesponsive to barrier tissue disruption and keratinocyte damage.

Together, our data demonstrate that obesity and metabolic disease negatively impact the homeostasis and wound healing functions of γδ T cells located in the epidermal barrier. The impact of chronic TNFα on γδ T cells was reversible, suggesting that therapeutic strategies targeting the inflammatory environment and γδ T cell dysfunction may provide additional treatments for complications associated with obesity, metabolic disease and type 2 diabetes. In addition to the skin, intraepithelial γδ T cells reside in multiple barrier tissue locations, including the lung and intestinal tract, and the impact of metabolic disease on the function of other resident γδ T cell populations is unknown. The consequence of reduced numbers and unresponsiveness of γδ T cells in multiple barrier tissues would result in compromised ability to protect against damage or environmental insults and increased susceptibility to infection. This study demonstrates a previously unrecognized biphasic progression of skin γδ T cell dysfunction in obesity and metabolic disease, in which hyperglycemia impacts skin γδ T cell proliferation and homeostasis and chronic inflammatory mediators alter skin γδ T cell response to barrier damage.

## Materials and Methods

### Ethics Statement

All animals were handled in strict accordance with good animal practice as defined by the relevant national and/or local animal welfare bodies. All animal work was approved by The Scripps Research Institute Institutional Animal Care and Use Committee (protocol 08-0057).

### Mice

Wild-type C57BLKS/J, BKS-*Lepr^db^* heterozygous (C57BLKS/J *db/+*), and B6- *Lepr^db^* heterozygous (C57BL/6J *db/+*) mice were purchased from The Jackson Laboratory (Bar Harbor) and were housed and bred at The Scripps Research Institute (TSRI). Wild-type C57BL/6J mice were bred at TSRI Rodent Breeding Colony. For high fat diet experiments, wild-type male C57BL/6J mice were placed on a 60 kcal% fat diet (Research Diets) at 6 weeks of age, control mice were maintained on a 5 kcal% (Harlan Laboratory) or a 10 kcal% (Research Diets) diet. To generate δ*^−/−^ db/db* mice, C57BL/6J δ*^−/−^* were crossed with C57BL/6J *db/+* mice to generate δ*^−/−^ db/+* mice. All mice were periodically weighed and blood glucose monitored by an Ascensia Elite XL blood glucose monitor (Bayer). BKS *db/+* and *db/db* mice were assayed at 6 weeks and at 10- and 14-weeks of age. For HFD experiments, mice were assayed after 20 to 26 weeks on HFD. Mice were given access to food and water ad libitum and were housed in sanitized conditions.

### Flow cytometry

FITC-, PE-, or allophycocyanin-conjugated monoclonal antibodies specific for γδ TCR (GL3), Vγ3^+^ (536), CD25 (PC61), TNFα (MP6-XT22), CD45.2, IL-4Rα and Thy1.2 (53.2.1) were purchased from BD Biosciences, CD69 (H1.2F3), CD103, CD3ε, and Langerin antibodies were purchased from eBioscience, and TGFβ1 was purchased from R&D Systems. The BD Bioscience Cytofix/Cytoperm kit was used for intracellular staining and Annexin-V/PI kit for flow cytometry. Cells were acquired with DiVa 5.0 software on a Digital LSRII (BD Biosciences) and analyzed with FlowJo software (Tree Star, Inc.). For FACS plots, gating was determined for each individual experiment using negative or isotype controls.

### *In vitro* γδ 7–17 cell line

The skin γδ T-cell line 7–17 was maintained in complete RPMI (Mediatech, Inc.) supplemented with 10% heat-inactivated FBS and 20U/ml IL-2. For proliferation studies, 7–17 cells were plated at 1×10^5^ cells per well in a 96 well flat bottom plate in IL-2 containing growth media with either glucose (MediaTech) or fatty acids (palmitic acid, oleic acid and linoleic acid (Sigma)). Cells were pulsed with 1 µCi/well [^3^H]thymidine (MP Biomedicals), harvested and incorporation of radioactive material was determined using a β-counter (Beckman). Fatty acids were prepared for cell culture assays as described elsewhere [55]. For analysis of phosphorylated STAT5A and STAT5B, 7–17 cells were pre-treated with starvation media for 4 hours, then placed into IL-2 containing growth media supplemented with 33.3 mM glucose for 24 hours. Cells were starved for additional 4 hours +/− glucose, followed by treatment with 40U/ml IL-2. Cells were lysed in TritonX Lysis Buffer, analyzed by Western blot using antibodies against phosphorylated STAT5A/STAT5B (Tyr^694^) and total STAT5 (Cell Signaling), probed with secondary goat anti-rabbit IgG-HRP (Southern Biotech) and developed with Super Signal West Pico Chemiluminescence Kit (Thermo Scientific).

### Epidermal cell preparation

Epidermal cells were isolated from mouse skin as described previously [1], [37] and rested at 37°C for 3–16 hours followed by antibody staining and flow cytometric analysis. For *in vitro* stimulation experiments, epidermal cells were isolated from mouse epidermis and placed into culture in complete DMEM media (Mediatech, Inc.) supplemented with 10% heat-inactivated FBS (Omega Scientific) and stimulated overnight with pre-coated anti-CD3ε antibody at 1 µg/ml. Approximately 16 to 18 hours after culturing, cells were treated with 5 µg/ml brefeldin A (Sigma) for 4 hours at 37°C, isolated and stained intracellularly with antibodies for flow cytometry. All cells were cultured at 37°C and 5% CO_2_.

### Freshly isolated γδ T cells

Epidermal cell preparations were prepared from wild-type C57BL/6J mice as described above, and γδ T cells were sorted on a FACSAria (TSRI Flow Cytometry Lab) based on anti-Thy1.2 antibody staining to a minimum of 95% purity. Skin γδ T cells were collected into FCS, spun down and placed directly into 96 well round bottom plates in normal growth media (cRPMI with 10% FBS and 100U/ml IL-2) with baseline (11.1 mM) or elevated (33.3 mM) glucose. Cell proliferation was based on [^3^H] thymidine incorporation as described above.

### BrdU treatment *in vivo*

Mice were given a one time i.p. injection of 3.3 mg/ml BrdU (Sigma) in PBS, followed by 7 days of BrdU in their drinking water at 0.8 mg/ml. Mice were euthanized on day 8 and epidermal cells were isolated as described above. BrdU incorporation was detected by using the FITC BrdU Flow Kit (BD Biosciences).

### Epidermal ear sheet and whole skin immunofluorescence

Epidermal sheets were isolated and stained as described previously [1], [37]. For *in vitro* stimulation assays, ears were removed from control *db/+* animals, separated in half and floated on DMEM media (supplemented with 10% FBS) and treated with 10 µg/ml anti-CD3ε antibody or 100 ng/ml recombinant TNFα (R&D Systems) as indicated. After indicated incubation at 37°C, ear sheet halves were removed, the epidermal sheet was separated from the dermis using ammonium thiocyanate and staining was performed. To visualize cross-sections of mouse skin, whole skin tissue was embedded in O.C.T. compound (Tissue-Tek), and 10 µm skin sections were cut on a Leica Cryostat. Sections were fixed with 4% paraformaldehyde for 10 minutes, and immunostained with γδ TCR and CD3ε antibodies. DAPI was used to counterstain the sections. Digital images were acquired (Zeiss AxioCam HRc) and analyzed using Photoshop CS2 software (Adobe). At least three separate experiments were performed for each time point and a minimum of 500 cells were quantified per experiment.

### Animal dorsal and ear wounding protocols

Full-thickness biopsy punch wounds were performed on the dorsal surface and ears of mice as previously described [1], [14]. At the indicated time after wounding, mice were euthanized and wounds were harvested. Epidermal sheets were isolated for analysis of γδ T cell rounding at the wound site by immunofluorescent microscopy. Epidermal cells were isolated using trypsin as described above, allowed to rest three hours in the presence of 5 µg/ml brefeldin A at 37°C, and followed by intracellular antibody staining for TNFα and/or TGFβ1 expression and analysis by flow cytometry. For anti-TNFα treatment, obese *db/db* animals were randomly assigned to a treatment group, then weighed and blood glucose determined before the start of experiment. Mice received 1 mg/kg either anti-TNFα or IgG control antibody (Biolegend) each day i.p. for a minimum of 4 days total. On the final day of treatment, mice were euthanized and full-thickness punch biopsy wounds were administered as described above. Non-wounded skin and skin at the wound edge was removed 24 hours post-wounding and epidermal cells were isolated and stained for intracellular cytokine production as described above.

### Microarray analysis

Epidermal cell preparations from mice were isolated and skin γδ T cells were sorted on a FACSAria as described above. Skin γδ T cells were collected directly into TRIzol LS reagent (Invitrogen), RNA was immediately isolated using the Qiagen RNeasy Micro RNA Kit and submitted to the TSRI DNA Array Core. 100 ng product was processed with GeneChip Whole Transcript Sense Target Labeling Assay (Affymetrix) and cDNA was hybridized overnight to the Mouse Gene 1.0ST Array (two independent data sets for each sample). Chips were scanned using the Affymetrix GeneChip Scanner 3000 7G with default settings and a target intensity of 250 for scaling. Data normalization was performed using RMA Express 1.0 with quantile normalization, median polish and background adjustment. This data has been deposited in NCBI's Gene Expression Omnibus and is accessible through GEO Series accession number GSE22196.

### PCR determination of Lep and Lepr isoforms (Ob-Ra and Ob-Rb)

RNA was isolated from primary sorted skin γδ T cells or 7–17 cell lines with TRIzol reagent and transcribed into cDNA with reverse transcriptase (Invitrogen). 1 µl cDNA was amplified using PCR with primers directed against Ob-Ra and Ob-Rb for 35 cycles [56], leptin for 30 cycles [57] and β-actin controls [1]. A plasmid containing leptin cDNA was kindly provided by Dr. Luc Teyton (The Scripps Research Institute, La Jolla).

### Lymph node staining

Lymph nodes were isolated from mice and pooled. Cells were mechanically disrupted from the tissue by gently agitating between two frosted slides in DMEM. Cells were stained for flow cytometric analysis using antibodies listed above.

### Statistic analysis

Data are presented as mean ± SEM or mean ± SD and significance was determined using the *t-test* function of Microsoft Excel (two-tailed).

## Supporting Information

Table S1 (0.04 MB DOC)

Figure S1 Expression of Leptin and Leptin Receptor in skin γδ T cells. RT-PCR for expression of Lep mRNA in skin γδ T cells isolated from BKS db/+ and db/db mice, or from skin γδ 7-17 T cells ±1 µg/ml anti-CD3ε stimulation for 2 hours or 24 hours. Shown is Lep cDNA positive control and H2O negative control. Expression of Lepr isoforms, Ob-Ra and Ob-Rb, mRNA was not detected in mouse γδ T cells. RT-PCR for expression of Lepr in skin γδ T cells isolated from BKS db/+ and db/db mice, or in 7–17 skin γδ T cells ±1 µg/ml anti-CD3ε stimulation for 2 hours or 24 hours. Shown is whole liver positive control and H2O negative control. β-actin expression was used to control for all PCR reactions.
(0.34 MB TIF)

Figure S2 Fatty acids do not inhibit skin γδ T cell growth. Proliferation of skin γδ 7–17 T cells in IL-2 containing growth media supplemented with palmitic, lineolic and oleic acid between 0 and 200 µM. Each experiment was performed in duplicate, data presented as mean ± SD.
(0.28 MB TIF)

Figure S3 Skin γδ T cells in the db/db mouse are not undergoing apoptosis or migration. (A) Multiparameter flow cytometry of annexin-V/PI staining of skin γδ T cells, gated on Thy1.2+ expression, at 6-, 8- and 14-weeks of age. Numbers indicate the percent of γδ T cells. A minimum of two experiments were performed per time point, shown is one representative experiment. (B) Skin sections from 10- to 14-week old BKS db/+ and db/db mice were immunostained with γδ TCR (red) and dapi (blue). Three separate experiments were performed with similar results. Magnification is ×200, bar represents 0.05 µm. (C) γδ T cell populations in skin-draining lymph nodes isolated from 10- to 14-week old BKS db/+ and db/db animals. In the upper plots, live cells were gated on Thy1.2+ and Vγ3+, exclusive markers for skin-specific γδ T cells. In the lower plots, cells were gated on γδ TCR+ and CD3+ T cells to visualize the peripheral γδ T cell population. Numbers indicate percent γδ T cells. Data are representative of two independent experiments.
(1.08 MB TIF)

Figure S4 Skin γδ T cell activation marker and γδ TCR expression is not altered by hyperglycemia. (A) Multiparameter flow cytometry of CD69, CD25 and CD103 on the cell surface of γδ T cells isolated from BKS db/+ and db/db in mice at 6-weeks of age. Numbers in the top right corners indicate percent of γδ T cells. (B) γδ TCR expression on γδ T cells isolated from BKS db/+ (solid line) and db/db (shaded gray) at 6-weeks of age. Dotted lines represent unstained controls. Epidermal cells were gated on live Thy1.2+ to distinguish γδ T cells. A minimum of three experiments were performed per age, shown is one representative experiment for each, the same number of events is presented for each dot plot.
(0.42 MB TIF)
