# Supplementary material for: γδ T Cells Are Reduced and Rendered Unresponsive by Hyperglycemia and Chronic TNFα in Mouse Models of Obesity and Metabolic Disease
Source: PLoS One. 2010 Jul 2;5(7):e11422. doi: 10.1371/journal.pone.0011422 (PMC2896399; doi:10.1371/journal.pone.0011422)
Supplement: Table S1 — (0.04 MB DOC) [file pone.0011422.s001.doc]

Supplemental Table 1. Mouse serum glucose levels and total body weight.

|  | **Glucose (mg/dl)** | |  | **Weight (grams)** | |
| --- | --- | --- | --- | --- | --- |
| **C57BLSK/J (BKS):** | **BKS *db/+*** | **BKS *db/db*** |  | **BKS *db/+*** | **BKS *db/db*** |
| 6-week old ♀ | 128 ± 4.9 | 160 ± 24.4 |  | 18 ± 0.3 | 27 ± 1.5 |
| 9-10-week old ♀ | 118 ± 3.3 | 355 ± 24.2 |  | 21 ± 0.8 | 39 ± 2.0 |
| 11-12-week old ♀ | 118 ± 7.2 | 333 ± 27.7 |  | 22 ± 1.0 | 42 ± 1.2 |
| 13-14-week old ♀ | 126 ± 7.1 | 354 ± 39.4 |  | 22 ± 1.1 | 46 ± 1.6 |
|  |  |  |  |  |  |
| **C57BL/6J (B6):** | **B6 *db/+*** | **B6 *db/db*** |  | **B6 *db/+*** | **B6 *db/db*** |
| 10-15-week old ♂ | 143 ± 9.4 | 314 ± 52.7 |  | 26.8 ± 0.3 | 42 ± 2.7 |
|  |  |  |  |  |  |
| **C57BL/6J (B6):** | **B6 NCD** | **B6 HFD** |  | **B6 NCD** | **B6 HFD** |
| 26-32-week old ♂ | 136 ± 9.4 | 178 ± 4.9 |  | 27.4 ± 0.8 | 53.0 ± 1.1 |

Data presented as mean ± s.e.m. ♀, female; ♂, male. For BKS *db/+* and *db/db* mice, a minimum of 3 mice were assayed per age. For B6 *db/+* and *db/db* and B6 NCD (normal chow diet) and HFD (high fat diet) mice, *n = 3*.
