# Supplementary figures and images for: γδ T Cells Are Reduced and Rendered Unresponsive by Hyperglycemia and Chronic TNFα in Mouse Models of Obesity and Metabolic Disease
Source: PLoS One. 2010 Jul 2;5(7):e11422. doi: 10.1371/journal.pone.0011422 (PMC2896399; doi:10.1371/journal.pone.0011422)

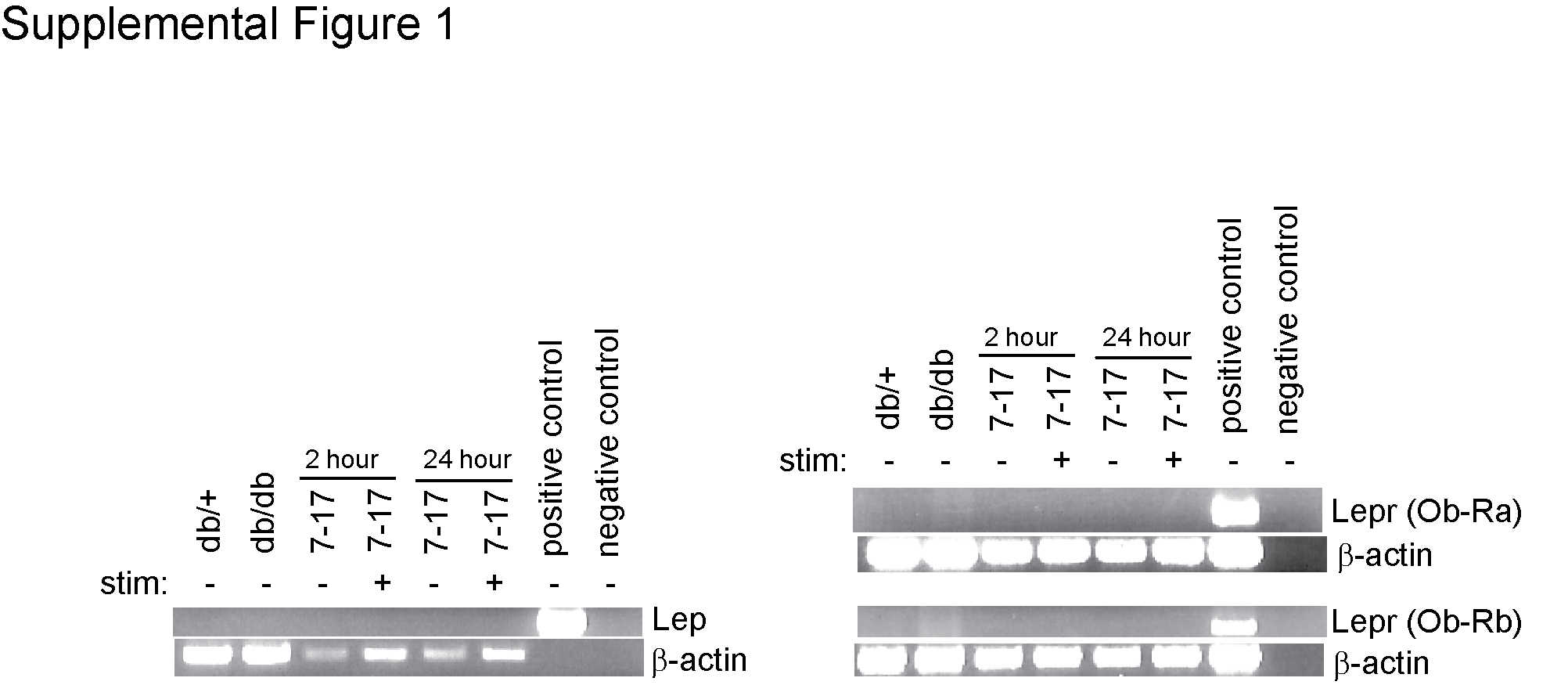

Supplement: Figure S1 — Expression of Leptin and Leptin Receptor in skin γδ T cells. RT-PCR for expression of Lep mRNA in skin γδ T cells isolated from BKS db/+ and db/db mice, or from skin γδ 7-17 T cells ±1 µg/ml anti-CD3ε stimulation for 2 hours or 24 hours. Shown is Lep cDNA positive control and H2O negative control. Expression of Lepr isoforms, Ob-Ra and Ob-Rb, mRNA was not detected in mouse γδ T cells. RT-PCR for expression of Lepr in skin γδ T cells isolated from BKS db/+ and db/db mice, or in 7–17 skin γδ T cells ±1 µg/ml anti-CD3ε stimulation for 2 hours or 24 hours. Shown is whole liver positive control and H2O negative control. β-actin expression was used to control for all PCR reactions. (0.34 MB TIF) [file pone.0011422.s002.tif]

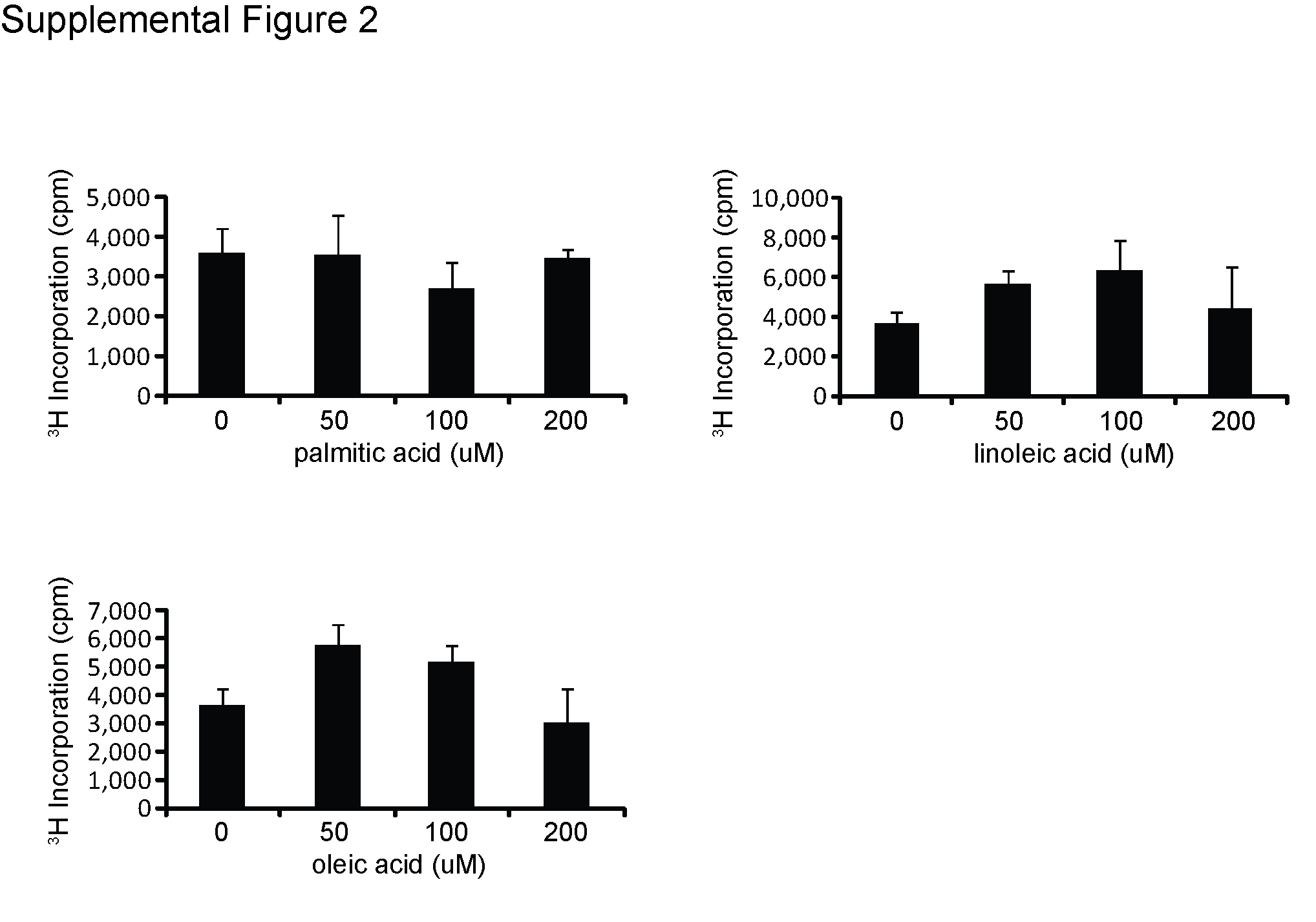

Supplement: Figure S2 — Fatty acids do not inhibit skin γδ T cell growth. Proliferation of skin γδ 7–17 T cells in IL-2 containing growth media supplemented with palmitic, lineolic and oleic acid between 0 and 200 µM. Each experiment was performed in duplicate, data presented as mean ± SD. (0.28 MB TIF) [file pone.0011422.s003.tif]

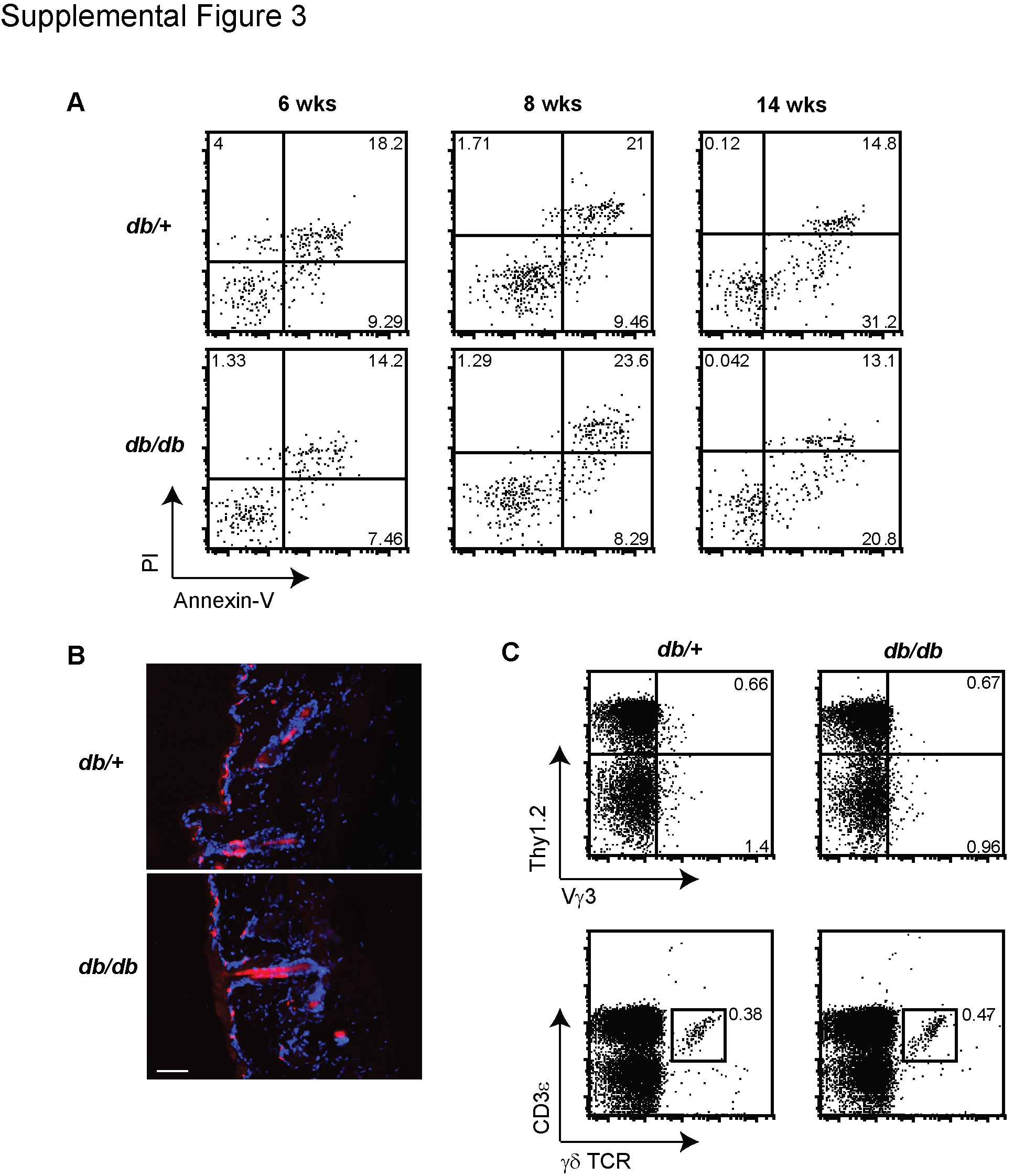

Supplement: Figure S3 — Skin γδ T cells in the db/db mouse are not undergoing apoptosis or migration. (A) Multiparameter flow cytometry of annexin-V/PI staining of skin γδ T cells, gated on Thy1.2+ expression, at 6-, 8- and 14-weeks of age. Numbers indicate the percent of γδ T cells. A minimum of two experiments were performed per time point, shown is one representative experiment. (B) Skin sections from 10- to 14-week old BKS db/+ and db/db mice were immunostained with γδ TCR (red) and dapi (blue). Three separate experiments were performed with similar results. Magnification is ×200, bar represents 0.05 µm. (C) γδ T cell populations in skin-draining lymph nodes isolated from 10- to 14-week old BKS db/+ and db/db animals. In the upper plots, live cells were gated on Thy1.2+ and Vγ3+, exclusive markers for skin-specific γδ T cells. In the lower plots, cells were gated on γδ TCR+ and CD3+ T cells to visualize the peripheral γδ T cell population. Numbers indicate percent γδ T cells. Data are representative of two independent experiments. (1.08 MB TIF) [file pone.0011422.s004.tif]

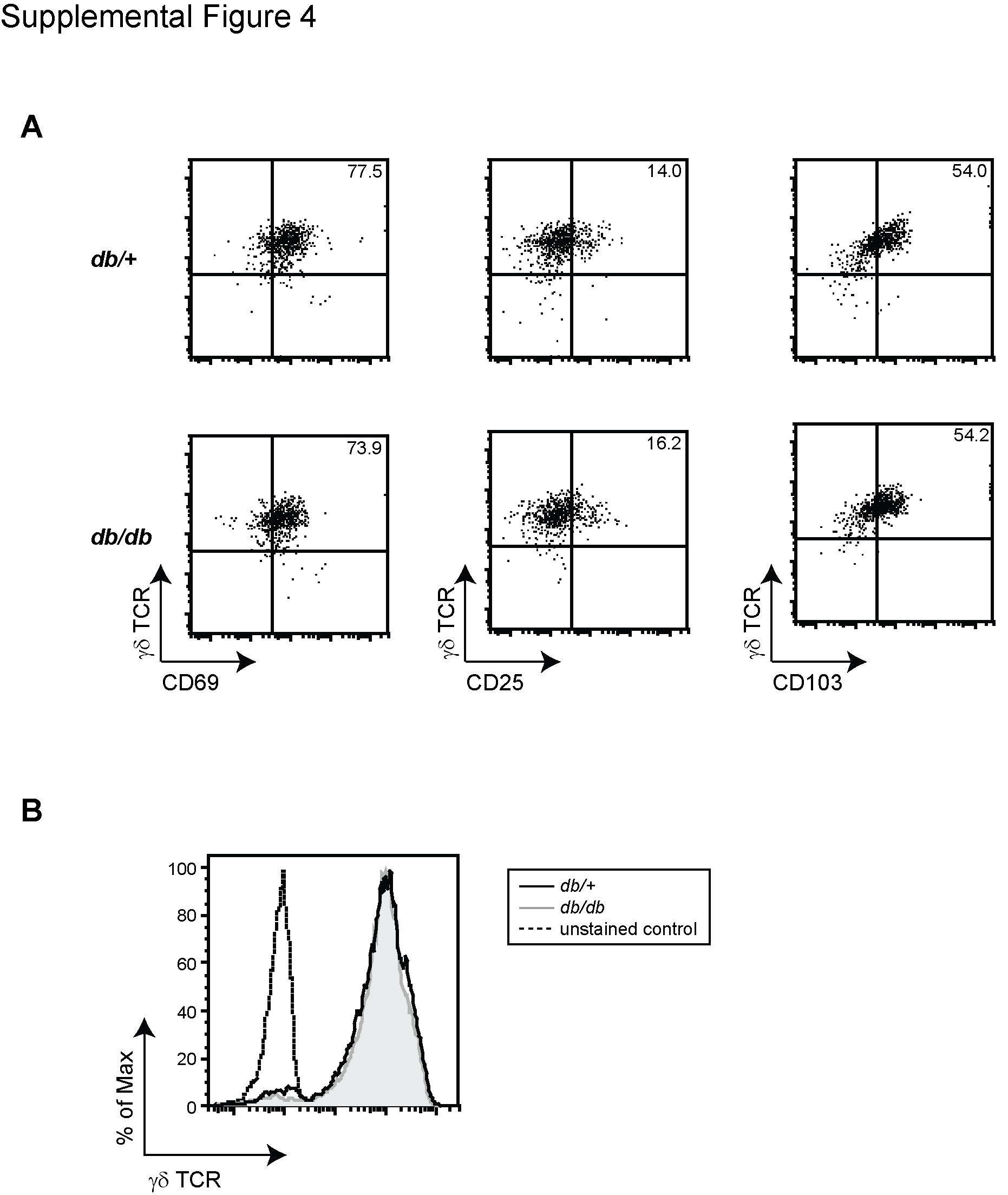

Supplement: Figure S4 — Skin γδ T cell activation marker and γδ TCR expression is not altered by hyperglycemia. (A) Multiparameter flow cytometry of CD69, CD25 and CD103 on the cell surface of γδ T cells isolated from BKS db/+ and db/db in mice at 6-weeks of age. Numbers in the top right corners indicate percent of γδ T cells. (B) γδ TCR expression on γδ T cells isolated from BKS db/+ (solid line) and db/db (shaded gray) at 6-weeks of age. Dotted lines represent unstained controls. Epidermal cells were gated on live Thy1.2+ to distinguish γδ T cells. A minimum of three experiments were performed per age, shown is one representative experiment for each, the same number of events is presented for each dot plot. (0.42 MB TIF) [file pone.0011422.s005.tif]
